# Enhanced Inhibition of Bladder Cancer Cell Growth by Simultaneous Knockdown of Antiapoptotic Bcl-xL and Survivin in Combination with Chemotherapy

**DOI:** 10.3390/ijms140612297

**Published:** 2013-06-07

**Authors:** Doreen Kunze, Kati Erdmann, Michael Froehner, Manfred P. Wirth, Susanne Fuessel

**Affiliations:** Department of Urology, University Hospital “Carl Gustav Carus”, Technische Universität Dresden, Fetscherstrasse 74, 01307 Dresden, Germany; E-Mails: kati.erdmann@uniklinikum-dresden.de (K.E.); michael.froehner@uniklinikum-dresden.de (M.F.); urologie@uniklinikum-dresden.de (M.P.W.); susanne.fuessel@uniklinikum-dresden.de (S.F.)

**Keywords:** apoptosis, BCL2L1, Bcl-xL, bladder cancer, BIRC5, chemotherapy, combination therapy, RNA interference, siRNA, survivin

## Abstract

The overexpression of antiapoptotic genes, such as Bcl-xL and survivin, contributes to the increased survival of tumor cells and to the development of treatment resistances. In the bladder cancer cell lines EJ28 and J82, the siRNA-mediated knockdown of survivin reduces cell proliferation and the inhibition of Bcl-xL sensitizes these cells towards subsequent chemotherapy with mitomycin C and cisplatin. Therefore, the aim of this study was to analyze if the simultaneous knockdown of Bcl-xL and survivin might represent a more powerful treatment option for bladder cancer than the single inhibition of one of these target genes. At 96 h after transfection, reduction in cell viability was stronger after simultaneous inhibition of Bcl-xL and survivin (decrease of 40%–48%) in comparison to the single target treatments (decrease of 29% at best). Furthermore, simultaneous knockdown of Bcl-xL and survivin considerably increased the efficacy of subsequent chemotherapy. For example, cellular viability of EJ28 cells decreased to 6% in consequence of Bcl-xL and survivin inhibition plus cisplatin treatment whereas single target siRNA plus chemotherapy treatments mediated reductions down to 15%–36% only. In conclusion, the combination of simultaneous siRNA-mediated knockdown of antiapoptotic Bcl-xL and survivin—a multitarget molecular-based therapy—and conventional chemotherapy shows great potential for improving bladder cancer treatment.

## 1. Introduction

Bladder cancer (BCa) is the sixth most commonly diagnosed malignancy in Europe, with an estimated 139,500 new cases, and 51,300 BCa-related deaths in the year 2008 [1]. At initial diagnosis, about 70%–75% of the patients present with non-muscle invasive BCa while the remaining 25%–30% already have a muscle invasive disease [2,3]. Standard treatment for non-muscle invasive BCa is transurethral resection and adjuvant intravesical chemotherapy or immunotherapy [4]. In spite of these therapies, approximately 43% of the non-muscle invasive BCa will relapse—*i.e*., tumors of the same stage and grade as the primary BCa will occur—and about 8% will progress to muscle invasive disease [2]. Patients with muscle invasive BCa will be treated by radical cystectomy [3]. Cisplatin-based combination chemotherapy is the standard treatment for metastatic urothelial bladder cancer. Although combination chemotherapy produces high response rates, and may even be curative in individual cases, overall, long-term survival rates are dismal [3]. Therefore, new strategies are needed to overcome resistance to chemotherapy.

Apoptosis, an essential biological process that mediates the maintenance of tissue homeostasis as well as the non-inflammatory elimination of damaged cells, is frequently deregulated in cancer [5]. Notably, the up-regulation of antiapoptotic genes contributes to the increased survival of tumor cells. Previous studies have shown that in BCa cells particularly the inhibition of the antiapoptotic factor survivin (=baculoviral IAP repeat containing 5, BIRC5) mediated direct antiproliferative effects such as reductions in cell viability [6,7]. Furthermore, the knockdown of Bcl-xL (=BCL2-like 1, BCL2L1) sensitized BCa cells to subsequent chemotherapy [8].

Survivin is a member of the inhibitor of apoptosis (IAP) family that is overexpressed in almost all human cancers, including BCa [9,10]. In contrast, survivin is not expressed in most terminally differentiated cells [9]. Survivin executes its antiapoptotic function by interactions with other proteins, e.g., the caspase inhibitor XIAP (X-linked inhibitor of apoptosis), thereby increasing the activity of the binding partners [11]. For instance, the expression of survivin in BCa tissue of cystectomy patients is associated with disease-specific mortality [10].

Bcl-xL, an antiapoptotic member of the BCL2 family, prevents caspase activation by inhibiting cytochrome c release from the mitochondria [12]. Bcl-xL expression in BCa samples is associated with elevated tumor stage and grade [13].

In this study, we present data showing that simultaneous knockdown of Bcl-xL and survivin in combination with subsequent chemotherapy is most effective in inhibiting BCa cell proliferation and therefore might represent a promising treatment option for BCa.

## 2. Results and Discussion

Two siRNAs were used against each target gene. The siRNAs targeting Bcl-xL were named “BX-A” and “BX-B” and the siRNAs targeting survivin were “S-A” and “S-B”. Simultaneous knockdown of Bcl-xL and survivin was analyzed by two siRNA combination treatments (“M2-A” and “M2-B”). In the M2-A combination the siRNAs BX-A and S-A were incubated simultaneously. BX-B and S-B were applied together in the M2-B combination (further information is given in the experimental section).

### 2.1. Reduction of Bcl-xL and Survivin Expression after siRNA Transfection

**Figure 1:**
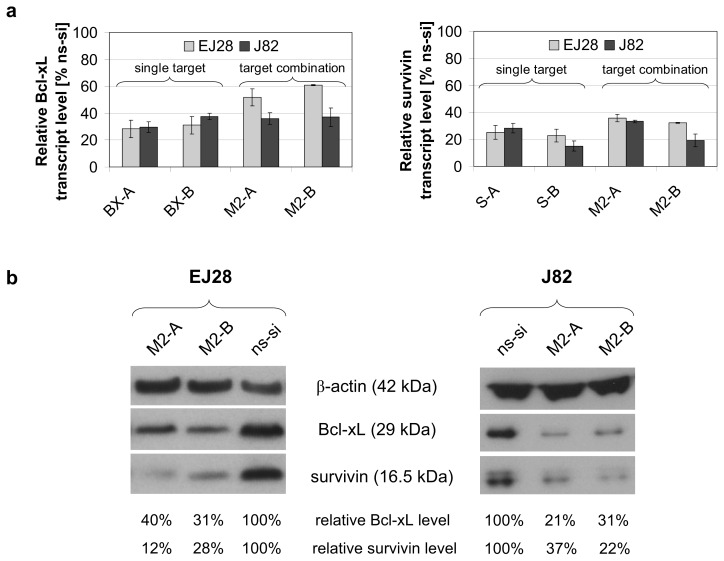
Effects of siRNA transfection on the expression of Bcl-xL and survivin. (**a**) Relative mRNA expression levels of Bcl-xL and survivin in EJ28 and J82 bladder cancer cells, 48 h after transfection. Expression values are normalized to the reference gene TBP and are shown relative to the control siRNA “ns-si” (=100%). Values represent averages of two independent experiments with their mean deviation; (**b**) Bcl-xL and survivin protein content detected by Western Blotting 48 h after transfection. Bcl-xL and survivin levels are shown normalized to the reference protein β-actin and relative to the ns-si control.

The selected target-specific siRNAs (40 nM) potently decreased the mRNA expression levels of Bcl-xL and survivin by 62%–85% in EJ28 and J82 BCa cells 48 h after transfection (Figure 1a). In the combination treatments M2-A and M2-B, in which both targets were inhibited simultaneously with 20 nM siRNA per target, transcript level reductions by 39%–81% were achieved (Figure 1a). Western Blot analysis verified Bcl-xL and survivin protein knockdown 48 h after siRNA transfection in both BCa cell lines (Figure 1b).

### 2.2. Cellular Effects of siRNA-Mediated Inhibition of Bcl-xL and Survivin

**Figure 2:**
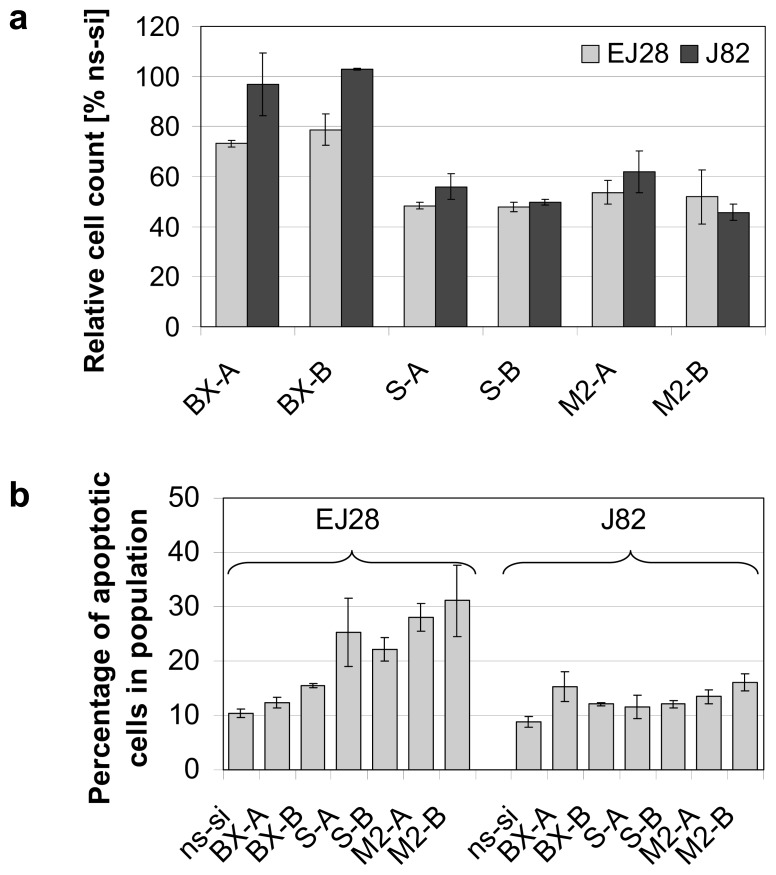
Reduction in cell counts and induction of apoptosis after single and combined siRNA-mediated inhibition of Bcl-xL and survivin in EJ28 and J82 bladder cancer cells. Analyses were performed 48 h after transfection. (**a**) Cell counts are shown relative to the control siRNA “ns-si” (=100%). Values shown are averages of two independent experiments with their mean deviation; (**b**) Percentage of apoptotic cells presented as sum of early and late apoptotic cells. Values shown are representatives of two independent experiments.

Strong reductions in cell counts, down to 46% compared to the control, were observed after knockdown of survivin as well as after simultaneous inhibition of Bcl-xL and survivin 48 h after transfection (Figure 2a). Apoptosis induction was highest after combined knockdown of both antiapoptotic genes. For example, a 2.7- to 3.0-fold increase in percentage of apoptotic cells was seen in the M2-A and M2-B treatments in EJ28 cells (Figure 2b). This is an increase in apoptosis rate from 10.4% in the ns-si control to 28.1% (M2-A) and to 31.1% (M2-B). In J82 cells, enhancement of apoptosis rate was less prominent. However, percentage of apoptotic cells in population increased from 9% in the ns-si control to up to 16% after M2-B treatment (Figure 2b). The effect of combined siRNA-mediated knockdown of Bcl-xL and survivin on cancer cell apoptosis was additive in EJ28 cells.

**Figure 3:**
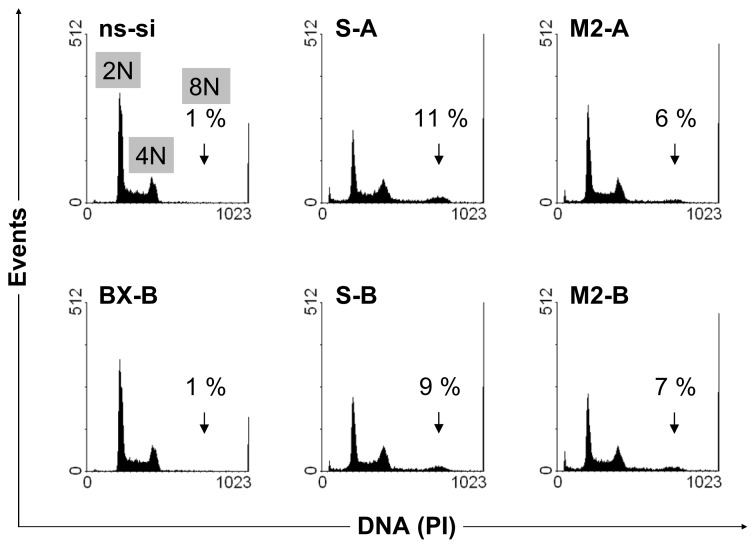
Induction of polyploidy after knockdown of survivin in single target and target combination treatments. DNA content of EJ28 cells, 48 h after single and combined siRNA-mediated inhibition of Bcl-xL and survivin, is shown. Arrows point at polyploid cells with DNA content of 8N. Representative images of two independent experiments are shown.

**Figure 4:**
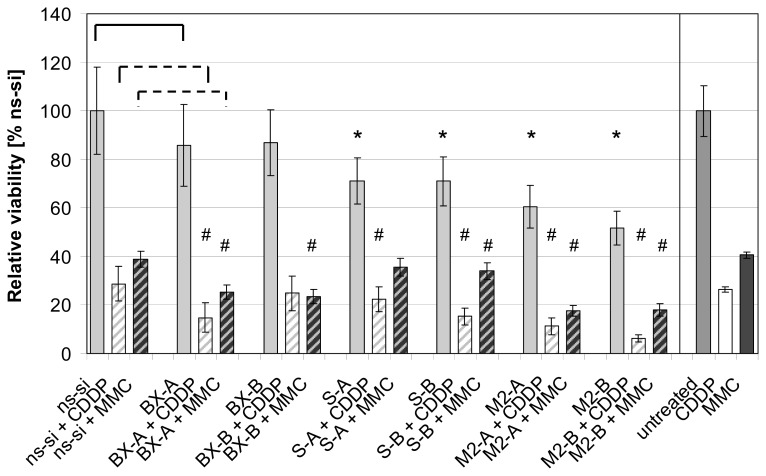
Reductions in viability of EJ28 cells after siRNA-mediated inhibition of Bcl-xL and survivin, with or without subsequent chemotherapy (CT). EJ28 cells were transfected with a total of 40 nM siRNAs for four hours. Twenty-four hours after transfection start, cells were treated with 2.1 μg/mL cisplatin (CDDP) for 24 h or with 0.9 μg/mL mitomycin C (MMC) for two hours. Cell viability was examined 96 h after transfection. Values shown are relative to the control siRNA “ns-si” (=100%, for all siRNA treatments) or relative to untreated cells (only for CDDP and MMC single treatments) and are averages of a fourfold determination. Error bars represent the 95% confidence interval. An unpaired Student’s *t*-test was used to compare the differences in cell viability between target-specific siRNA and ns-si treated cells (*p ≤ 0.05) as well as between target-specific siRNA + CT and ns-si + CT treated cells (^#^*p* ≤ 0.05).

No changes in cell cycle distribution were found in both BCa cell lines after Bcl-xL knockdown (exemplarily shown for BX-B treatment in EJ28 cells, Figure 3) whereas inhibition of survivin, as well as combined knockdown of both target genes, caused polyploidy (Figure 3). For example, 6% and 7% of the EJ28 cells showed DNA content of 8N after transfection with the siRNA combinations M2-A and M2-B. Viability of EJ28 cells was significantly reduced by 29% in consequence of survivin knockdown, 96 h after siRNA transfection (Figure 4). Even stronger, simultaneous inhibition of Bcl-xL and survivin mediated cell viability reductions of 40% and 48% (Figure 4). An even greater impact on cell viability—40% and 48% reduction—was observed after simultaneous inhibition of Bcl-xL and survivin (Figure 4).

### 2.3. Effects of Combined Treatment with siRNAs and Chemotherapy

**Figure 5:**
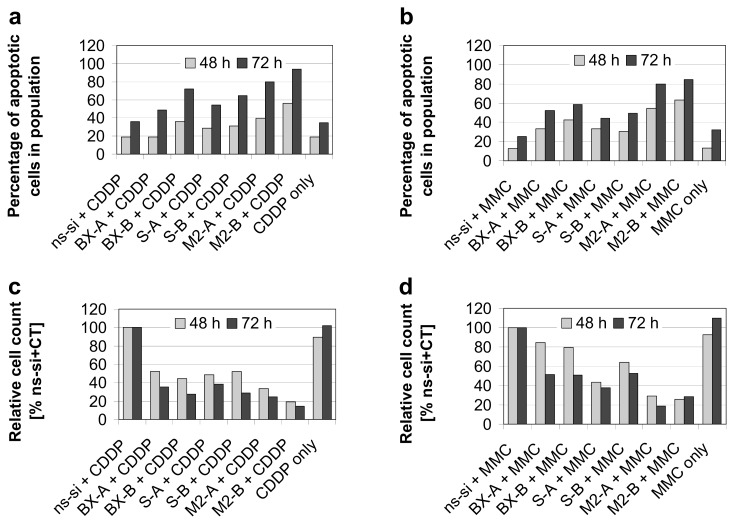
Induction of apoptosis and reduction in cell counts after combined siRNA plus chemotherapy (CT) treatments in EJ28 bladder cancer cells. Cells were transfected with the respective siRNAs for four hours. “CT only” cells were treated with serum-free OptiMEM medium during transfection. Twenty-four hours after transfection start, cells were treated with 2.1 μg/mL cisplatin for 24 h (**a**,**c**) or with 0.9 μg/mL mitomycin C for 2 h (**b**,**d**). Rate of apoptosis—presented as sum of early and late apoptotic cells—(**a**,**b**), as well as cell counts (**c**,**d**) were determined 48 h and 72 h after transfection start.

**Figure 6:**
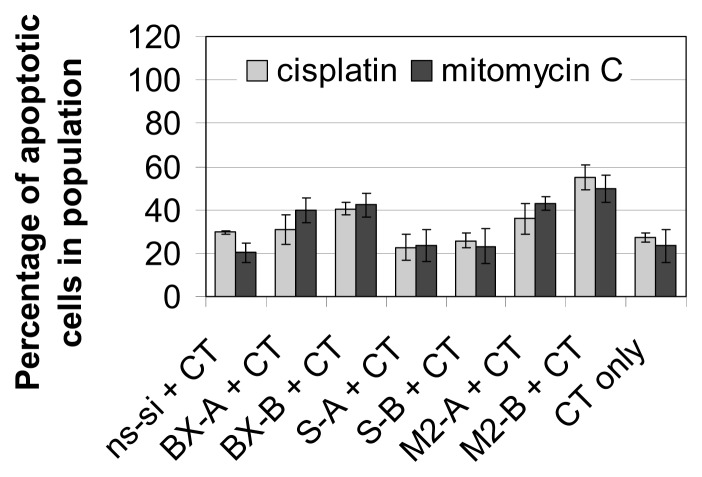
Apoptosis rate after treatment of J82 bladder cancer cells with siRNAs and chemotherapy (CT). Cells were transfected with the respective siRNAs for four hours. “CT only” cells were treated with serum-free OptiMEM medium during transfection. Twenty-four hours after transfection start, cells were treated with 1.2 μg/mL cisplatin for 24 h or with 1.0 μg/mL mitomycin C for two hours. Apoptosis rate—presented as sum of early and late apoptotic cells—was determined 72 h after transfection start. Values shown are averages of two independent experiments. Error bars represent the mean deviation.

As shown previously, knockdown of Bcl-xL notably increased efficacy of cisplatin and mitomycin C therapy in EJ28 BCa cells [8]. For example, while inhibition of Bcl-xL alone only marginally increased apoptotic rate from 10% in the control to 12%–16% (Figure 2b), 33%–43% of the cells were apoptotic after BX-A or BX-B plus mitomycin C treatment compared to 13% in the ns-si + mitomycin C control (Figure 5b). Strongest enhancement of chemotherapy efficacy is mediated by simultaneous knockdown of Bcl-xL and survivin. In EJ28 cells, cellular viability decreased down to 6% after M2-B + cisplatin treatment, whereas single target siRNA + chemotherapy treatments mediated reductions down to only 15%–36% (Figure 4). In addition, the lowest number of cells, as well as the highest percentage of apoptotic cells in population, were found after M2-A and M2-B treatments in combination with subsequent chemotherapy in both BCa cell lines (Figures 5 and 6). For example, 94% of the EJ28 cells were apoptotic in consequence of M2-B + cisplatin treatment, 72 h after transfection (Figure 5a).

**Figure 7:**
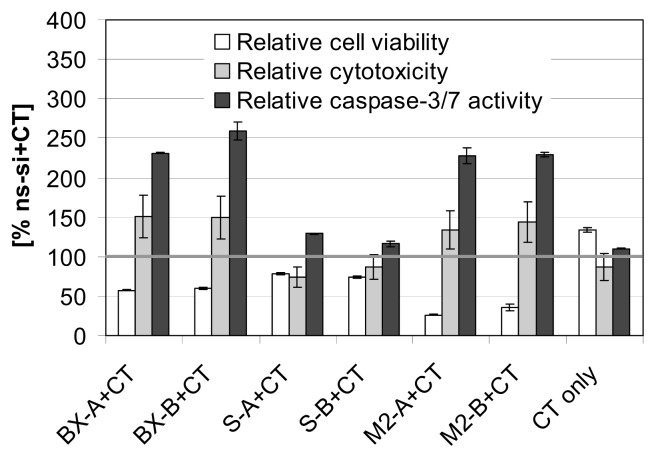
Analyses of cell viability, cytotoxicity and caspase-3/7 activity after treatment of EJ28 cells with siRNAs and subsequent chemotherapy (CT). Cells were transfected with the respective siRNAs for four hours. “CT only” cells were treated with serum-free OptiMEM medium during transfection. Twenty-four hours after transfection start, cells were treated with 0.9 μg/mL mitomycin C for two hours. ApoTox-Glo triplex assay was performed 72 h after transfection. Values shown are mean values of duplicates and are relative to the control siRNA “ns-si” plus CT treatment (=100%).

The ApoTox-Glo Triplex Assay, which was performed exemplarily with EJ28 cells and the chemotherapeutic mitomycin C, verified reductions in cell viability mediated by Bcl-xL knockdown as well as by simultaneous inhibition of both antiapoptotic genes together with subsequent mitomycin C treatment (Figure 7). Moreover, this assay showed strong enhancement of caspase-3/7 activity in these samples, e.g., a 2.3-fold increase in caspase-3/7 activity after M2-A and M2-B plus chemotherapy treatment, and in consequence an increase of cytotoxicity (Figure 7).

### 2.4. Discussion

Deregulation of programmed cell death, apoptosis, is a hallmark of cancer cells [5]. Antiapoptotic members of the BCL2 family as well as the members of the IAP family, which impair the execution of cell death processes, are often overexpressed in human cancers, including BCa, and enhance tumor cell survival [10,13,14]. Previous studies have shown that the knockdown of antiapoptotic BCL2 (B-cell CLL/lymphoma 2) and XIAP did not alter growth characteristics of the BCa cell lines EJ28 and J82, whereas inhibition of survivin decreased BCa cell proliferation and reductions in Bcl-xL levels increased chemotherapy efficacy [6–8]. Therefore, the aim of this study was to analyze, if the simultaneous knockdown of Bcl-xL and survivin might be superior in eliminating BCa cells to the single inhibition of these targets.

Our results show that the combined inhibition of Bcl-xL and survivin caused BCa cell growth inhibitory effects comparable to single survivin knockdown at 48 h after transfection (Figure 2a). However, 96 h after treatment, reductions in cell viability were strongest after simultaneous inhibition of both antiapoptotic factors (Figure 4). Furthermore, targeting of Bcl-xL and survivin at the same time increased the effects of anticancer chemotherapy more pronounced than single target inhibitions. The tumor cell growth-inhibiting effects of Bcl-xL plus survivin knockdown and subsequent chemotherapy were stronger than after simultaneous inhibition of the four antiapoptotic genes BCL2, Bcl-xL, XIAP and survivin plus chemotherapy (Figures 4 and 5, and [8]). This is presumably due to the minor importance of BCL2 and XIAP in the analyzed BCa cell lines, and to the increased antiproliferative effects of survivin knockdown in the M2-A and M2-B treatments with 20 nM per siRNA in comparison to the previously conducted treatments in which BCL2, Bcl-xL, XIAP, and survivin were inhibited simultaneously by 10 nM siRNA per target gene [8]. For example, 6%–7% of the BCa cells showed a DNA content of 8N after M2-A and M2-B treatment whereas only 2% did after simultaneous inhibition of BCL2, Bcl-xL, XIAP and survivin (Figure 3 and [6]). This induction of polyploidy is mediated by reduction in cellular survivin levels because survivin, an integral part of the chromosomal passenger complex, also regulates chromosome segregation and cytokinesis [15]. The impairment of mitosis and cytokinesis is not related to increased levels of apoptosis in the analyzed EJ28 and J82 BCa cells. While induction of polyploidy after survivin knockdown was observed in both BCa cell lines, apoptosis induction occurred only in EJ28 cells (Figure 2b). Formation of polyploidic cells after survivin knockdown by siRNAs or antisense oligonucleotides was shown previously in different types of cancer [7,16–18].

The knockdown of genes that contribute to tumor development and progression shows great potential as an anticancer strategy [19]. Furthermore, the overexpression of certain genes, e.g., genes encoding drug efflux transporters such as ABCB1 (also known as MDR1), proteins of the human nucleotide excision repair such as ERCC1 as well as antiapoptotic proteins, has been shown to interfere with the efficacy of chemotherapy [20,21]. Likewise, high levels of specific proteins, e.g., IAP proteins and the regulator of cell cycle progression cyclin D1, can mediate resistance of cancer cells towards radiotherapy [22,23]. Therefore, the combination of target-specific molecular-based treatments with chemotherapy or radiotherapy could increase the efficacy of the conventional therapies and reverse acquired treatment resistances. The contribution of each target to malignant proliferation or to treatment resistance needs to be evaluated specifically for each tumor entity to omit unsuitable target genes while selecting the most relevant ones and in consequence to obtain the best possible therapies with minimal side effects. For example, the antiapoptotic proteins BCL2 and XIAP are of minor importance in EJ28 and J82 BCa cells, while Bcl-xL and survivin seem to be most important [6]. However, in other tumor entities, such as gastric cancer and non-small cell lung cancer, siRNA-mediated knockdown of BCL2 and XIAP, respectively, induced apoptosis and reduced tumor growth [24,25]. Likewise, even though the phosphatidylinositol 3-kinase (PI3K) p110 isoforms α and β are both upregulated in paclitaxel-resistant ovarian cancer cells only the siRNA-mediated knockdown of PI3K p110β resensitized these cells towards paclitaxel [26].

Since the inhibition of a single target might be easily bypassed by cancer cells, e.g., by upregulation of other tumor growth-promoting genes [27,28], a multitarget approach seems to be more promising. As well, in the combination therapy settings where target-specific inhibitors and conventional treatments such as chemotherapy and radiotherapy were applied together, the simultaneous knockdown of carefully selected genes can be superior to single target inhibition. For example, the apoptotic rate in T24 BCa cells was significantly higher after simultaneous knockdown of livin, XIAP, and survivin in combination with mitomycin C than after single target knockdown plus chemotherapy [29]. As well, simultaneous targeting of two of the three antiapoptotic targets BCL2, Bcl-xL, and XIAP increased radiosensitivity of chondrosarcoma cells to a greater extent than the single inhibition of one gene [30]. Di Cresce *et al.* showed that simultaneous siRNA-mediated inhibition of thymidylate synthase and thymidine kinase 1 or 2 sensitized HeLa cells to 5-fluorodeoxyuridine and pemetrexed [31]. PC-3 prostate cancer cells were sensitized towards TRAIL treatment by combined inhibition of cIAP-1, cIAP-2, and XIAP, but not by single target knockdown [32].

The successful delivery of the negatively charged siRNA constructs *in vivo* remains the major challenge for clinical siRNA application [33]. Particularly, the systemic administration of siRNAs provides several problems such as nuclease-mediated siRNA degradation, kidney filtration, as well as transport across the vascular endothelial barrier and the uptake into the target cells [34]. A study by Davis *et al.* showed that these obstacles can be overcome. The authors proved RRM2 target gene inhibition in tumor tissue after successful systemic siRNA application using a targeted, nanoparticle delivery system in patients with solid cancers [35]. For non-muscle invasive BCa, a possible application is the instillation of siRNAs—also together with a chemotherapeutic—after transurethral resection of the tumor. This local application should avoid many problems of a systemic siRNA delivery. The applicability of this approach was already shown in an orthotopic BCa mouse model. The intravesical treatment with liposome-encapsulated siRNAs targeting survivin and PLK1 successfully reduced the mRNA levels of the targets and reduced tumor growth [36].

## 3. Experimental Section

### 3.1. Cell Culture

The human BCa cell lines EJ28 (University of Frankfurt, Frankfurt, Germany) and J82 (ATCC, Manassas, VA, USA)—both derived from muscle invasive bladder cancers—were cultured in Dulbecco’s modified Eagle’s medium (4.5 g/L glucose) containing 10% fetal calf serum, 1% MEM non-essential amino acids and 1% HEPES (all from Life Technologies, Darmstadt, Germany) under standard conditions (37 °C, humidified atmosphere containing 5% CO_2_).

### 3.2. siRNA Transfection

Two siRNAs against each target were selected and synthesized by Eurogentec (Seraing, Belgium). The siRNA target sequences were CAGCUGGAGUCAGUUUAGU (=BX-A) as well as GGGACAGCAUAUCAGAGCU (=BX-B) for Bcl-xL, and GAAGCAGUUUGAAGAAUUA (=S-A) as well as CCAACAAUAAGAAGAAAGA (=S-B) for survivin. All siRNAs had 3′-dTdT overhangs. Twenty-four or 72 h after seeding, cells were transfected for 4 h in serum-free OptiMEM (Life Technologies, Darmstadt, Germany) with a total of 40 nM siRNAs using DOTAP liposomal transfection reagent (ratio 1:30, *w*/*w*) according to the manufacturer’s instructions (Roche, Mannheim, Germany). In the siRNA combination “M2-A” the siRNAs BX-A and S-A (20 nM each) were incubated simultaneously. Accordingly, the constructs BX-B and S-B (20 nM each) were used in the treatment “M2-B”. Cells were transfected with 40 nM of the negative control siRNA (ns-si, reference: SR-CL000-005, Eurogentec) as control and for normalization. After 4 h, transfection medium was replaced by fresh culture medium and cells were incubated for a further 20 h to 92 h. For analyses, cells were harvested by trypsin treatment (0.05% trypsin/0.02% EDTA, 5 min, 37 °C). Detached and adherent cells were pooled and analyzed together.

### 3.3. Treatment with Chemotherapy

Cisplatin and mitomycin C were used as chemotherapeutics. Cisplatin-based combination chemotherapy is standard in the treatment of metastatic bladder cancer and might also be used in patients with localized muscle-invasive tumors before or after cystectomy [3]. Mitomycin C is applied as adjuvant intravesical chemotherapy after transurethral resection of non-muscle invasive bladder cancers [4]. Since the siRNAs might also act as chemosensitizers for improving the prevention of recurrence and progression of non-muscle invasive bladder cancers, we also analyzed if the selected siRNAs are able to increase mitomycin C efficacy. This was done as preliminary test using the EJ28 and J82 bladder cancer cell lines that are derived from muscle invasive tumors.

If cells were treated with chemotherapeutics after siRNA transfection the following protocol was applied. Firstly, cells were transfected as described above. Secondly, chemotherapeutics were added 24 h after the start of transfection. Final concentrations of cisplatin and mitomycin C were 2.1 μg/mL and 0.9 μg/mL, respectively, for EJ28 cells. For J82 cells, 1.2 μg/mL cisplatin and 1.0 μg/mL mitomycin C were used. Cisplatin was incubated for 24 h, and mitomycin C for 2 h. Following this, cells were washed once with PBS and were further cultivated with fresh culture medium. The ns-si + chemotherapy combination was used as control to evaluate the siRNA-mediated effects of the treatment.

### 3.4. Cell Counts and Cell Viability

Cells were counted using the Coulter Z2 Particle Count & Size Analyser (Beckman Coulter, Krefeld, Germany). Examination of cellular viability was performed in quadruplicates using the cell proliferation reagent WST-1 (Roche, Mannheim, Germany). Cells were seeded into 96-well culture plates and treated with siRNAs with or without subsequent chemotherapy as described above. After treatment, cells were incubated with 100 μL fresh culture medium. At 96 h after transfection start, 10 μL WST-1 reagent were added to the cells. After incubation for 1–3 h, absorbance was measured with a spectrophotometer (Anthos labtec, Krefeld, Germany) at 450 nm and at 620 nm as reference.

### 3.5. Apoptosis Detection and Cell Cycle Analyses

Apoptosis was assessed by annexin V/propidium iodide (PI) staining, 48 h and 72 h after transfection start, using flow cytometry (Annexin V-FITC Apoptosis Detection Kit I, BD Biosciences, Heidelberg, Germany). In brief, after treatment, 1 × 10^5^ cells were washed with cold PBS and resuspended in 100 μL binding buffer. Subsequently, 5 μL annexin V-FITC and 5 μL PI were added. Cells were incubated for 15 min at room temperature in the dark. After addition of a further 400 μL, binding buffer cells were analyzed by flow cytometry (FACScan, BD Biosciences, Heidelberg, Germany). Percentage of early (annexin V-FITC positive, PI negative) and late (annexin V-FITC positive, PI positive) apoptotic cells were determined by quadrant analysis of annexin V-FITC/PI plots using WinMDI2.8 software [37]. The CycleTest Plus DNA Reagent Kit (BD Biosciences, Heidelberg, Germany) was used according to the manufacturer’s instructions for cell cycle analysis, 48 h after transfection, by flow cytometry.

### 3.6. ApoTox-Glo Triplex Assay

The ApoTox-Glo Triplex Assay (Promega, Mannheim, Germany) enables the consecutive determination of cell viability, cytotoxicity, and apoptosis in the same sample. Cells were seeded into black 96-well culture plates with clear bottoms and were treated with siRNAs with or without subsequent chemotherapy as described above. After treatment, cells were incubated with 100 μL fresh cell culture medium. At 72 h after transfection start, 20 μL Viability/Cytotoxicity Reagent were added to the cells. Following incubation for 1 h, fluorescence was measured with the microplate multimode reader Mithras LB 940 (Berthold Technologies, Bad Wildbad, Germany) at 355_Ex_/495_Em_ for quantification of cell viability and at 485_Ex_/535_Em_ for determination of cytotoxicity. Afterwards, 100 μL Caspase-Glo 3/7 Reagent were added to each well. Following incubation for 30 min, luminescence was measeured with the microplate multimode reader.

### 3.7. RNA Isolation, cDNA Synthesis, and Quantitative PCR

Total RNA was isolated using the InviTrap Spin Cell RNA Mini Kit (Invitek, Berlin, Germany) according to the manufacturer’s instructions. Afterwards, RNA was reverse transcribed into cDNA (SuperScript II Reverse Transcriptase; Life Technologies, Darmstadt, Germany). Transcript amounts of Bcl-xL, survivin and the reference gene TBP (TATA box binding protein) were determined by quantitative real-time PCR (qPCR) using the primers, probes, and kits listed in Table 1.

### 3.8. Western Blot

Cells, 5 × 10^4^ per sample, were lysed in 20 μL loading buffer (20% glycerol, 2% SDS, 125 mM Tris pH 6.8, 5% β-mercaptoethanol, bromophenol blue). After incubation at 95 °C for 5 min, proteins were separated on 8%–16% Precise Protein Gels (Fisher Scientific, Schwerte, Germany) and transferred onto PVDF membranes (GE Healthcare, Freiburg, Germany). Membranes were incubated with primary antibodies against Bcl-xL (1:100; clone 2H12; QED Bioscience Inc., San Diego, CA, USA) or survivin (1:1,000; NB500-201; Novus Biologicals, Littleton, CO, USA). Beta-actin detected by a monoclonal anti-β-actin antibody (1:20,000; Sigma-Aldrich, St. Louis, MO, USA) served as a loading control. The secondary polyclonal swine anti-rabbit immunoglobulins HRP-linked antibody (1:1,000, Dako, Glostrup, Denmark) for survivin, and the polyclonal rabbit anti-mouse immunoglobulins HRP-linked antibody (1:1,000; Dako, Glostrup, Denmark) for Bcl-xL and β-actin as well as the Enhanced Chemiluminescence Kit (GE Healthcare, Freiburg, Germany) were used for visualization. Content of the respective proteins was quantified by densitometry using Quantity One Basic software (Bio-Rad Laboratories, Munich, Germany).

### 3.9. Statistics

Data are presented as mean ± mean deviation. Cell viability results are expressed as mean ± 95% confidence interval. An unpaired Student’s *t*-test was used to compare the differences in cell viability between cells treated with target-specific siRNAs *vs.* cells treated with ns-si (*p ≤ 0.05) as well as between cells treated with target-specific siRNAs + chemotherapy *vs.* cells treated with ns-si + chemotherapy (^#^*p* ≤ 0.05).

## 4. Conclusions

In conclusion, the simultaneous inhibition of multiple tumor-relevant genes in combination with conventional therapies—such as the siRNA-mediated knockdown of Bcl-xL plus survivin together with subsequent mitomycin C or cisplatin therapy—represents a promising option for improving bladder cancer treatment.

## Figures and Tables

**Table 1:** Sequences of primers and probes for quantitative PCR.

Target	Sequence 5′→3′
Bcl-xL 1	target-specific Real-Time Reagent Mix (AJ Roboscreen, Leipzig, Germany) containing the appropriate primers and probes
survivin^2^	Primers: for: GAACTGGCCCTTCTTGGAG, rev: AAGTCTGGCTCGTTCTCAGTG Probe: Universal ProbeLibrary Probe #86 (Roche, Germany, cat.no. 04689119001)
TBP 1	Primers: for: GAATATAATCCCAAGCGGTTTG, rev: ACTTCACATCACAGCTCCCC Probes: TTTCCCAGAACTGAAAATCAGTGCC-FL, LC-TGGTTCGTGGCTCTCTTATCCTCATG-PH

1 LightCycler FastStart DNA Master Hybridization Probes (Roche);

2 LightCycler TaqMan Master (Roche);

abbreviations: FL—fluorescence dye fluorescein; LC—fluorescence dye LC Red640; PH—phosphorylated 3′-end.
